# Selection Criteria for Determination of Optimal Reconstruction Method for Cu-64 Trastuzumab Dosimetry on Siemens Inveon PET Scanner

**DOI:** 10.3390/jcm8040512

**Published:** 2019-04-14

**Authors:** Seonhwa Lee, Hyeongi Kim, Ye-rin Kang, Hyungwoo Kim, Jung Young Kim, Yong-Jin Lee, Jung Min Kim, Jin Su Kim

**Affiliations:** 1 Division of RI Application, Korea Institute of Radiological and Medical Sciences (KIRAMS), 75 Nowon-ro, Nowon-gu, Seoul 01812, Korea; 2015010289@korea.ac.kr (S.L.); hyeongi@kirams.re.kr (H.K.); jykim@kirams.re.kr (J.Y.K.); yjlee@kirams.re.kr (Y.-J.L.); 2 Department of Bio-Convergence Engineering, Korea University, Seoul 02856, Korea; minbogun@korea.ac.kr; 3 Radiologicial and Medico-Oncological Sciences Major, University of Science and Technology (UST), Seoul 01812, Korea; kucs25@korea.ac.kr (Y.-r.K.); hungwoo.kim@khu.ac.kr (H.K.); 4 School of Health and Environmental Science, College of Health Science, Korea University, Seoul 02856, Korea; 5 College of Korean Medicine, Kyung Hee University, Seoul 02454, Korea

**Keywords:** Cu-64, trastuzumab, dosimetry, NEMA NU4-2008 guideline, Inveon PET

## Abstract

The goal of this study was to suggest criteria for the determination of the optimal image reconstruction algorithm for image-based dosimetry of Cu-64 trastuzumab PET in a mouse model. Image qualities, such as recovery coefficient (RC), spill-over ratio (SOR), and non-uniformity (NU), were measured according to National Electrical Manufacturers Association (NEMA) NU4-2008. Mice bearing a subcutaneous tumor ($200{\;\mathrm{mm}}^{3}$, HER2 NCI N87) were injected with monoclonal antibodies (trastuzumab) with Cu-64. Preclinical mouse PET images were acquired at 4 time points after injection (2, 15, 40 and 64 h). Phantom and Cu-64 trastuzumab PET images were reconstructed using various reconstruction algorithms (filtered back projection (FBP), 3D reprojection algorithm (FBP-3DRP), 2D ordered subset expectation maximization (OSEM 2D), and OSEM 3D maximum a posteriori (OSEM3D-MAP)) and filters. The absorbed dose for the tumor and the effective dose for organs for Cu-64 trastuzumab PET were calculated using the OLINDA/EXM program with various reconstruction algorithms. Absorbed dose for the tumor ranged from 923 mGy/MBq to 1830 mGy/MBq with application of reconstruction algorithms and filters. When OSEM2D was used, the effective osteogenic dose increased from 0.0031 to 0.0245 with an increase in the iteration number (1 to 10). In the region of kidney, the effective dose increased from 0.1870 to 1.4100 when OSEM2D was used with iteration number 1 to 10. To determine the optimal reconstruction algorithms and filters, a correlation between RC and NU was plotted and selection criteria (0.9 < RC < 1.0 and < 10% of NU) were suggested. According to the selection criteria, OSEM2D (iteration 1) was chosen for the optimal reconstruction algorithm. OSEM2D (iteration 10) provided 154.7% overestimated effective dose and FBP with a Butterworth filter provided 20.9% underestimated effective dose. We suggested OSEM2D (iteration 1) for the calculation of the effective dose of Cu-64 trastuzumab on an Inveon PET scanner.

## 1. Introduction

Immuno-PET uses monoclonal antibodies (mAbs) that are labeled with positron-emitting radionuclides. Immuno-PET can be applied for tailored patient management, dose calculation in radioimmunotherapy (RIT), and development of antibody-based drugs [1,2,3]. Cu-64 decays by both β+ (655 keV, 17.4%) and β− (573 keV, 39.0%) emission, making it suitable for both immuno-PET and radioimmuno-therapy (RIT) [4]. One of the widely used clinical trials was Cu-64 trastuzumab [5,6,7]. The safety of Cu-64 trastuzumab in HER2-positive breast cancer has been investigated using radiation dosimetry [8].

Image-based radiation dosimetry in nuclear medicine provides information on the absorption of energy from ionizing radiation and on the effect of radiation damage to living tissues. Image-based dosimetry could be used to assess the safety and efficacy of immuno-PET [9,10,11,12]. For the implementation of image-based dosimetry, the cumulative activity (Ã) should be calculated. The Ã is the product of the amount of activity present in the source organ and the length of time during which this activity is present [11,12]. To calculate the amount of cumulative activity, the region of interest (ROI) was drawn on the reconstructed PET or SPECT data. The dimensions of the matrix, the zoom factors, the spatial filters, and the reconstruction algorithms are factors that determine the spatial resolution of PET [13].

Performance measurement studies using the National Electrical Manufacturers Association (NEMA) standard NU 4-2008 [14] for a preclinical scanner provided us with information on spatial resolution, sensitivity, and image quality, such as non-uniformity and recovery coefficients [14,15,16,17]. In addition, a comparative study regarding reconstruction algorithms with filters was performed in terms of spatial resolution, recovery coefficient, and spill-over ratio, according to the NEMA standard NU4 [18,19,20]. Comparative studies for quantification of Zr-89 and I-124 were also performed [18,19,20,21]. However, there are no results from the investigation of reconstruction algorithms when various reconstruction algorithms were applied to the dosimetry study. 

In this study, we investigated the effect of reconstruction algorithms on the result of image-based dosimetry using Cu-64 trastuzumab PET on a Siemens Inveon PET scanner. In addition, we suggested criteria for the determination of the optimal reconstruction algorithms. To our best knowledge, our investigation is the first paper on selection criteria for determination of the optimal reconstruction algorithm and the relationship between the reconstruction algorithm and image-based radiation dosimetry on immuno-PET small animal studies.

## 2. Experimental Section

### 2.1. Ethical Statement

This article does not contain any studies involving human participants. The study was approved in accordance with institutional IACUC protocols.

### 2.2. PET Scanner 

All PET scans in this study were performed on a Siemens Inveon PET scanner (Siemens Medical Solutions, Tarrytown, NY, USA) [16].

### 2.3. Reconstruction Algorithms

All reconstruction algorithms implemented on the Inveon PET scanner, such as filtered back projection (FBP), 3D reprojection algorithm (FBP-3DRP), 2D ordered subset expectation maximization (OSEM 2D), and OSEM 3D maximum a posteriori (OSEM3D-MAP), were used for phantom and preclinical studies. 

For the FBP reconstruction, the ramp, Butterworth, Hamming, Hanning, Parzen, and Shepp–Logan filters were used. The cutoff frequency was 0.5. For OSEM2D algorithm, the subset number was 16 and the iteration numbers from 1 to 10 were used. OSEM3D is one of the most widely used reconstruction methods. When the iteration number was set to 0 during OSEM3D-MAP reconstruction, OSEM3D without MAP reconstruction method could be implemented on Inveon PET scanner. For the OSEM3D-MAP reconstruction, various smoothing factors (β) were used with MAP iteration number 18. MAP Iteration 18 was used on the basis of the result of Lasnon and colleagues. According to the results of Lasnon and colleagues (Figure 1 from ref. [18]), there was no significant change in the non-uniformity, recovery coefficient (RC), or spill-over ratio (SOR) with iteration number for the OSEM3D-MAP reconstruction. Both MAP and this implementation of OSEM3D contained information such as the scanner geometry and point spread function (PSF) of the detector response for the various angles that the 511 keV photons struck the detector. This information was not contained in any of the other reconstruction algorithms (FBP, 3DRP, and OSEM2D). The scanner geometry information was stored in the Pmatrix files, whereas the PSF information was stored in the blur kernels. MAP blur kernels were two-dimensional, whereas the OSEM3D blur kernels was one-dimensional, which led to better resolution recovery for MAP than for OSEM3D. In addition, MAP used a smoothing parameter (β value) that constrained neighboring voxels in the reconstructed image.

Siemens also provided “MAP iteration 18” as a default option instead of OSEM3D during OSEM3D-MAP reconstruction algorithm on Inveon PET scanner. Therefore, in this present study, we focused on the comparison of β smoothing factor during OSEM3D-MAP reconstruction.

**Figure A2:**
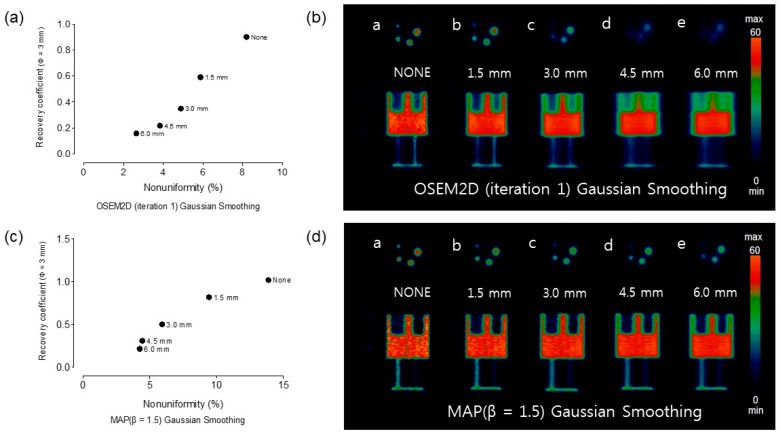
RC and NU of OSEM2D (iteration 1) and OSEM3D-MAP (β = 1.5) data with application of Gaussian smoothing kernel. Applied Gaussian FWHM was 1.5 mm, 3.0 mm, 4.5 mm, and 6.0 mm, respectively. (**a**) Plot of NU vs. RC (rod diameter, 3 mm) for OSEM2D (iteration 1), (**b**) representative PET data, (**c**) plot of NU vs. RC (rod diameter, 3 mm) for OSEM3D-MAP (β = 1.5), and (**d**) representative PET data. MAP: OSEM3D-MAP.

To measure the effect of post-smoothing with Gaussian kernels, various Gaussian smoothing kernels (1.5 mm, 3.0 mm, 4.5 mm, and 6.0 mm) were applied to reconstructed PET data. OSEM2D with iteration 1 and OSEM3D–MAP with β = 1.5 were representatively selected. The results of the effect of Gaussian smoothing were described in Figure A2 and Appendix A. We used Inveon reconstruction software suite (IAW version 1.4.3) for reconstruction. 

### 2.4. Attenuation and Scatter Corrections

We assessed the effects of attenuation correction (AC), and AC and scatter correction (SC) in PET. All emission data were acquired within an energy window of 350–650 keV. Transmission (TX) data were acquired using a Co-57 source (activity, 136 MBq) for 15 min. TX PET data were acquired within an energy window of 120–125 keV. Attenuation coefficients were scaled to 511 keV automatically during histogramming. Blank data were also acquired using a Co-57 source for 3 h. Normalization data were acquired for 10 h. All TX and blank data were histogrammed by single slice rebinning. All emission data and normalization data were sorted into a 3D sinogram. SC was performed for all datasets using a single scatter simulation (SSS) scatter correction algorithm. SC was implemented using Inveon reconstruction software suite (IAW version 1.4.3).

### 2.5. Phantom Studies

To determine the optimal reconstruction parameters for Cu-64 trastuzumab PET, phantom studies were performed according to the NEMA NU4 guidelines [14]. The recovery coefficient (RC), non-uniformity (NU), and spill-over ratio (SOR) were measured. Image quality phantom data for Cu-64 was acquired for 20 min within an energy window of ~350–650 keV. Radioactivity was 3.7 MBq. Normalization, AC, and SC were applied. The matrix size was 128 × 128 × 159 and the voxel size was 0.776 × 0.776 × 0.796 mm^3^. NU, RC, and SOR were calculated for data that were only corrected for normalization, as well as for data that were corrected for AC and AC and SC effects. In this study, the term “partially corrected” meant that normalization, dead-time, and random correction were performed.

#### 2.5.1. Non-Uniformity

To determine the NU, a cylindrical volume of interest (VOI) (length 10 mm and diameter 22.5 mm) was drawn at the center of a uniform region. Mean value and standard deviation (SD) were measured in VOI. Non-uniformity was expressed in percent SD (% SD: Percent of standard deviation divided by mean).

#### 2.5.2. Recovery Coefficient

The RC is a measure of how well a PET scanner system can recover the true radioactivity concentration in the corresponding uniform area. RC can be obtained as following: 

RC = measured peak activity concentration/true activity concentration. 

To determine the RCs, five fillable rods of a radioactive source (1, 2, 3, 4, and 5 mm in diameter) were used at the bottom of the cylinder (length: 20 mm, diameter: 30 mm). Circular ROIs were drawn around each rod. The size of the ROI was twice the physical diameter of the rods. 

#### 2.5.3. Spill-Over Ratio

The SOR was defined as the ratio of the mean value in each cold cylinder to the mean value in the uniform area. The upper part of the uniform region was a cold region consisting of two empty cylinders (length: 15 mm, inner diameter: 8 mm, outer diameter: 10 mm). One space was filled with air and the other space with nonradioactive water. Although both cylinders were nonradioactive, scattered coincidence and random coincidence contributed to activity in the nonradioactive cylinder. In these non-radioactive regions, SOR values could be calculated to determine the effect of scattered or random coincidences in the nonradioactive region. To calculate the SOR, 2 cylindrical VOIs (length: 7.5 mm, diameter: 4 mm) were drawn in the air- and water-filled compartments. 

### 2.6. Quantification of Cu-64 Trastuzumab PET

#### 2.6.1. Cell Culture and Tumor Xenograft in Mice

NCI-N87 HER2+ cancer cell lines, obtained from the American Type Culture Collection (ATCC), were maintained in Roswell Park Memorial Institute (RPMI) medium containing 10% fetal bovine serum (FBS) with antibiotics (Sigma, St. Louis, MO, USA) at 37 °C in a humidified 5% CO_2_ incubator. The NCI-N87 cells (5 × 106) were injected subcutaneously into the right flank of female athymic BALB/c nude mice (*n* = 5) aged 5 weeks (Shizuoka Laboratory Animal Center, Hamamatsu, Japan). Tumor size was measured using a digital caliper and tumor volume was calculated by applying the formula width^2^ × length × 0.4. All experiments were performed in accordance with the institutional guidelines of the Korea Institute of Radiological and Medical Sciences (KIRAMS). 

#### 2.6.2. Radiolabeling of Cu-64-DOTA-Trastuzumab

Trastuzumab (20 mg) was added to DOTA-NHS-ester (1 mg, Macrocyclics) in 800 μL of 1 M sodium bicarbonate buffer (pH 8.5) and mixed gently for 24 h at 4 °C. Unconjugated chelator was removed using PD-10 column with 1 mM sodium acetate (pH 5.5). Cu-64 was produced at KIRAMS by 50-MeV cyclotron irradiation. Trastuzumab (Herceptin; F. Hoffmann–La Roche, Basel, Switzerland) was labeled with Cu-64 by conjugation with DOTA. ^64^CuCl_2_ (370 MBq) was added to 1 mg of DOTA-trastuzumab in 1 mM sodium acetate (pH 5.5) and incubated for 1 h at 37 °C. Instant thin-layer chromatography on silica gel (solvent—citric acid) showed that the radiolabeling yield was ≥95%.

#### 2.6.3. Cu-64 Trastuzumab PET

Three to four weeks after the implantation of the tumor, the tumor size reached about 200 mm^3^; Cu-64 DOTA trastuzumab (400 μCi/150 μg) was injected intravenously through a tail vein. Mice were anesthetized with 2% isoflurane in 100% oxygen (Forane solution; ChoongWae Pharma, Seoul, Korea). PET data were acquired during 15 min at time points of 2 h, 15 h, 40 h, and 64 h after injection of Cu-64 trastuzumab. PET data were reconstructed using various algorithms and filters to compare their performance. 

For the acquisition of anatomical image, X-ray CT for mice was acquired with full rotation and 180 projections using the Inveon system. Exposure time was 200 ms, and the estimated scan time was for 504 s X-ray CT. X-ray CT data were reconstructed using Feldkamp reconstruction with Shepp–Logan filtering. Effective pixel size of the reconstructed X-ray CT image was 109.69 μm × 109.69 μm. X-ray CT was used for the delineation of ROI. ROIs were delineated in the regions of the brain, lungs, liver, stomach, intestines, kidney, and tumor. The size of the ROIs ranged from 0.0067 to 0.066 cm^2^. After delineation of ROIs on X-ray CT, ROIs were copied to Cu-64 trastuzumab PET data. The maximum value from the ROI region was extracted and the percentage of injected dose/gram (% ID/g) was calculated. 

#### 2.6.4. Dosimetry

The radiation dose per unit of administered activity (mSv/MBq), the effective dose in organ, and the absorbed dose for the tumor region on Cu-64 trastuzumab PET in mice were calculated using OLINDA/EXM software (OLINDA; Vanderbilt University, Nashville, TN, USA) [9]. Time activity curves (TACs) were obtained for each organ. Decay-uncorrected TACs were derived and cumulative activity was obtained from the area under the curve (AUC) for TACs. For each source organ, the residence time was calculated by dividing the cumulative activity by the total injected dose. Olinda used models for an average adult male or female human. Scaling of mouse-derived time activity data was applied before entering residence time data into Olinda. The scaling method was described in our previous work [10].

For the calculation of the absorbed dose in the tumor, a sphere model in OLINDA was used. Tumor volume was calculated on Cu-64 trastuzumab PET data with multiple slices of ROIs. Tumor mass was calculated under the assumption of 1 g/mL. The absorbed S-value for each tumor volume was calculated with scaling by mass. A non-linear fitting between the S-value and the mass was used, because linear interpolation could provide too large a value of S. 

The effective dose for each organ and the absorbed dose for the tumor region were calculated using various reconstruction algorithms and filters.

## 3. Results

### 3.1. Non-Uniformity

**Figure 1:**
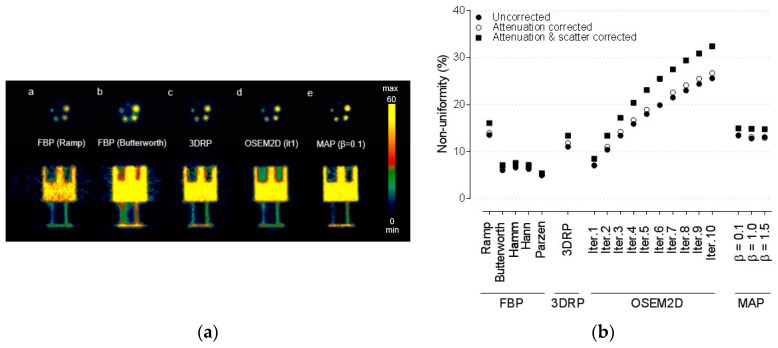
(**a**) Representative Cu-64 PET data of National Electrical Manufacturers Association (NEMA) NU4-2008 phantom. The reconstruction algorithms used were filtered back projection (FBP) (ramp filter), FBP (Butterworth filter), FBP-3DRP, 2D ordered subset expectation maximization (OSEM 2D) (iteration 1), and OSEM3D-MAP (β = 0.1). These data were presented for partially corrected data. (**b**) Non-uniformity for various reconstruction algorithms, such as FBP, FBP-3DRP, OSEM2D, and OSEM3D-MAP reconstruction with filters. Normalization, dead-time, and random corrections have been applied to all PET raw data. The effects of attenuation correction (AC), and AC and scatter correction (SC) corrections in PET were also provided. The term “partially corrected” meant that normalization, dead-time, and random correction were performed. AC: attenuation correction, SC: scatter correction, 3DRP: FBP-3DRP, MAP: OSEM3D-MAP.

Figure 1 shows the result of NUs, expressed in % SD, for various reconstruction algorithms and filters. The lowest NU (lowest % SD) was achieved when FBP with a Parzen filter was used. The values of NU were 5.00% for partially corrected, 4.95% for AC corrected, and 5.39% for AC and SC corrected, respectively. When OSEM2D reconstruction method was used, NUs increased with an increase of the iteration number. This means that images become noisier with increasing number of iterations. For analytical reconstruction methods such as FBP and FBP-3DRP, the differences in NUs between the methods were negligible (<3%) after AC was applied compared to partially corrected images. The NUs increased by 13% when AC and SC were applied. For iterative reconstruction methods, such as OSEM2D or OSEM3D-MAP reconstruction methods, the differences in NUs were within 5% between partially corrected images and AC images. When AC and SC were applied, the NUs increased by 23% for the OSEM2D reconstruction method. 

### 3.2. Recovery Coefficient and Non-Uniformity

**Figure 2:**
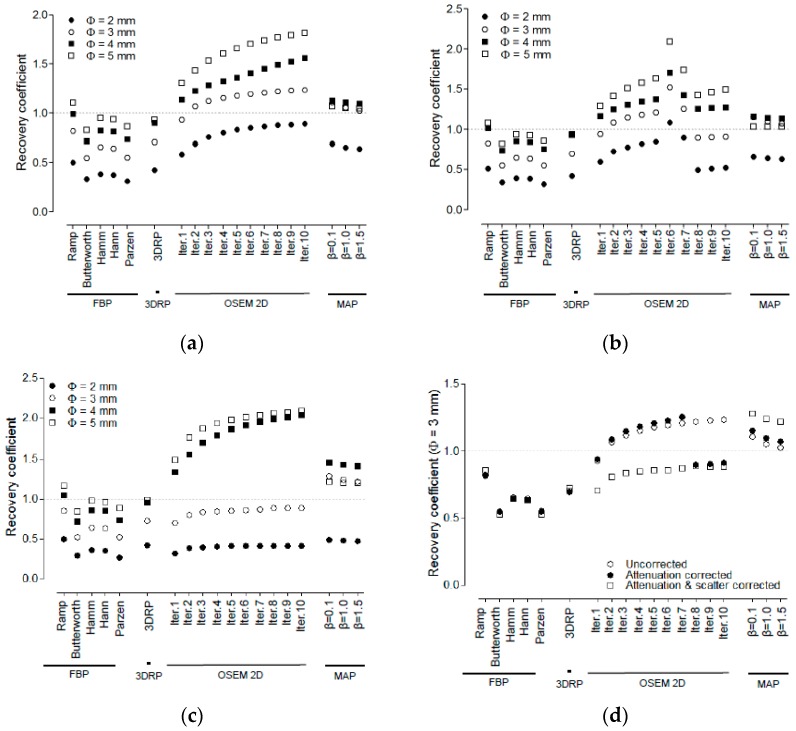
Recovery coefficient for various reconstruction algorithms, such as FBP, FBP-3DRP, OSEM2D, and OSEM3D-MAP reconstruction with filters. All data were presented for (**a**) partially corrected data, (**b**) AC, and (**c**) AC and SC data. The diameter of the rods were 2, 3, 4, and 5 mm. 1 mm diameter of rod was not visible. (**d**) RCs of 3 mm rods for various reconstruction methods. All data were presented for partially corrected data, AC and AC & SC data. 3DRP: FBP-3DRP, MAP: OSEM3D-MAP.

Figure 2a–c shows the RCs when partially corrected, with AC applied, and AC and SC applied, respectively. The RC analysis for rods with a size of 1 mm was not performed, because they were not discernible on the PET image. Figure 2d shows the RCs of 3 mm rods for various reconstruction methods when AC and SC were applied. When FBP was used with ramp filters for Cu-64 PET imaging, the highest RC values were 0.50, 0.86, 1.04, and 1.17 for 2, 3, 4, and 5 mm rods, respectively. Regarding the iterative reconstruction algorithms for Cu-64 PET imaging, the highest RC values were 0.42, 0.88, 2.04, and 2.09 for 2, 3, 4, and 5 mm rods when OSEM2D (iteration 10) was used. Regarding OSEM3D-MAP reconstruction algorithms, the highest RC values were 0.49, 1.29. 1.46, and 1.21 for 2, 3, 4, and 5 mm rods.

**Figure 3:**
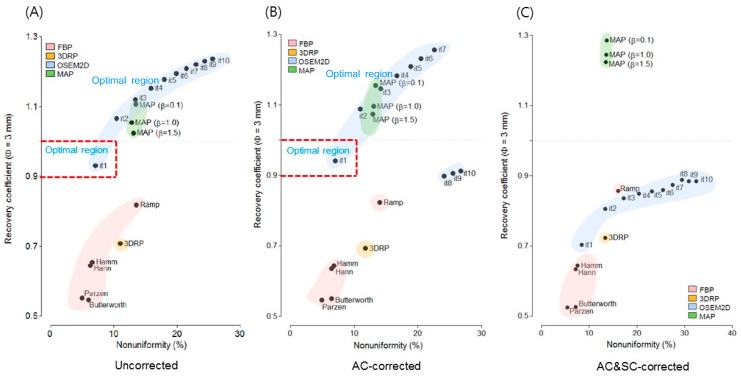
Plot of non-uniformity versus recovery coefficient (rod diameter is 3 mm) for various reconstruction algorithms, such as FBP, FBP-3DRP, OSEM2D, and OSEM3D-MAP reconstruction with filters. All data were presented for (**a**) partially corrected data, (**b**) AC corrected, and (**c**) AC and SC corrected data. The optimal criteria (0.9 < RC < 1.0 and NU < 10%) are marked in the red box. 3DRP: FBP-3DRP, MAP: OSEM3D-MAP.

Figure 3a–d shows the RC for 3 mm rods plotted against the NU when partially corrected, with AC, and AC and SC, respectively. OSEM2D (iteration 1) provided an RC value of 0.92 and an NU of 7.0% for partially corrected and OSEM2D (iteration 1) provided an RC value of 0.94 and an NU of 7.1% for AC. However, when AC & SC corrections were applied, the RC value deteriorated to 0.70 for OSEM2D (iteration 1). 

### 3.3. Spill-Over Ratio

**Figure 4:**
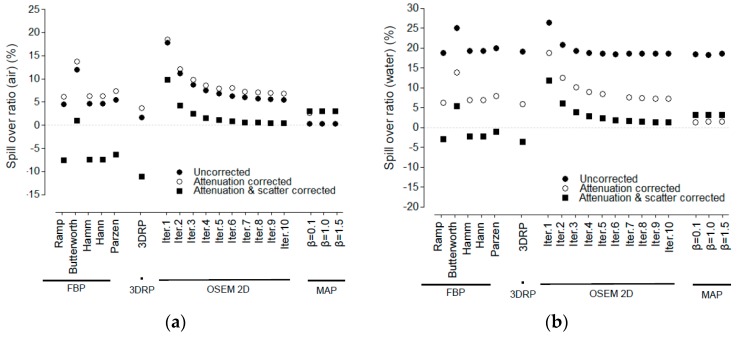
Spill-over ratio for various reconstruction algorithms, such as FBP, FBP-3DRP, OSEM2D, and OSEM3D-MAP reconstruction with filters. All data were presented for partially corrected data, AC corrected, and AC and SC corrected. Spill-over ratio for (**a**) air and (**b**) water. The term “partially corrected” meant that normalization, dead-time, and random correction were performed. 3DRP: FBP-3DRP, MAP: OSEM3D-MAP.

Figure 4 shows the SOR in the air and water compartments. For both the air and water compartments, FBP-3DRP provided the lowest SOR among the reconstruction algorithms for partially corrected, AC, and AC and SC. 

### 3.4. Cu-64 Trastuzumab PET

**Figure 5:**
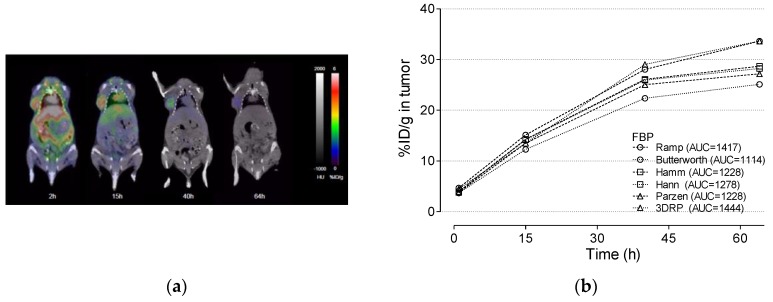

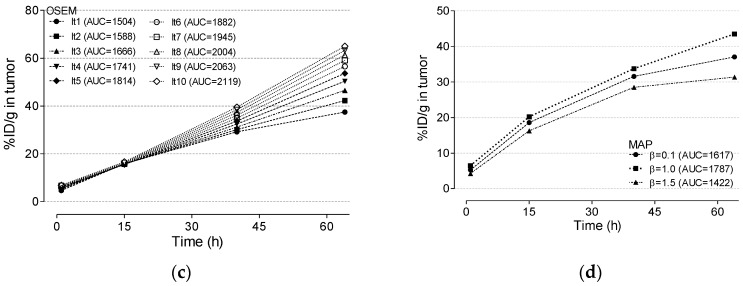
(**a**) PET image for 2, 15, 40, and 64 h after injection of Cu-64 trastuzumab. The PET data was overlaid on the X-ray CT data. % ID/g versus the time in the tumor region. % ID/g was calculated using various reconstruction algorithms, such as (**b**) FBP and FBP-3DRP, (**c**) OSE2D, and (**d**) OSEM3D-MAP. 3DRP: FBP-3DRP, MAP: OSEM3D-MAP.

Figure 5a shows the PET images of Cu-64 trastuzumab at 2, 15, 40, and 64 h after administration of Cu-64 trastuzumab using a HER2 + NCI N87 mouse model. Figure 5b shows the time activity curve and the corresponding AUC of the tumor region for various reconstruction algorithms. We confirmed there was no presence of negative values in the TAC, which was allowed for FBP and FBP-3DRP algorithms. The smoothing that was imposed by the OSEM3D-MAP algorithms would be the reason for the lower TAC values compared to OSEM2D. Lower values of TAC were obtained when a higher value of smoothing factor (β) was applied during MAP reconstruction.

### 3.5. Radiation Dosimetry Using Various Reconstruction Algorithms and Filters

Table 1 shows the results of radiation dosimetry for Cu-64 trastuzumab PET. The absorbed dose for the tumor ranged from 923 mGy/MBq to 1830 mGy/MBq. The absorbed dose for the tumor was 1200, 923, 1020, and 1020 mGy/MBq when FBP was used with ramp, Butterworth, Hamm, Hann, and Parzen filters, respectively. When OSEM2D was used, the absorbed dose to the tumor increased from 1320 to 1830 mGy/MBq with increasing number of iterations. Effective doses for organs have the same tendency. The effective dose for osteogenic was 0.0023 and 0.0047 when FBP reconstruction with Butterworth filter or Parzen filter was used, respectively. When OSEM2D was used, the effective dose for osteogenesis increased from 0.0031 to 0.0245 with increasing number of iterations. In the region of the kidney, the effective dose increased from 0.1870 to 1.4100 when OSEM2D was used.

## 4. Discussion

Radiation dosimetry provides information on the absorption of energy from ionizing radiation and the effect of radiation damage to living tissues during immuno-PET. Dosimetry can be used to provide data on the safety and efficacy of immuno-PET [9,10].

In this study, we investigated the effect of reconstruction algorithms and filters on radiation dosimetry for Cu-64 trastuzumab PET. For this purpose, image quality parameters, such as NU, RC, and SOR, were assessed for various reconstruction algorithms implemented in Siemens Inveon PET/CT. We suggested selection criteria for determining the optimal reconstruction algorithm and filter for the dosimetry study of Cu-64 trastuzumab PET.

### 4.1. Spill-Over Ratio

For both the air and water compartments, there were negative activity values in FBP or FBP-3DRP analytical algorithms but not in OSEM 2D or OSEM3D-MAP statistical iterative algorithms. FBP or FBP-3DRP could provide negative values because of ramp filtering [22]. The lowest SOR values were generally associated with the analytic algorithms because they can trigger negative background activity values and thus negative SOR scores. The OSEM-based OSEM2D and OSEM3D-MAP algorithms are characterized by the so-called non-negativity constraint and thus cannot yield negative SOR scores [23].

### 4.2. Selection of the Optimal Reconstruction Algorithm (a Trade-Off Relationship between Recovery Coefficients and Non-Uniformity)

To determine the optimal reconstruction algorithm, graphs between RC and NU were illustrated for partially corrected, AC, and both AC and SC data sets (Figure 3a–c). The factors that determine RC include the size of the lesion. The factors determining the spatial resolution in the image are the matrix size of the image, the image reconstruction method, and the filter [24]. The NU is an index that measures the uniformity of an image. RC can be considered as an index for the measurement of the peak value of a tumor. NU was the parameter for the assessment of the warm background around the tumor. The process of detecting a tumor in PET is the process of detecting a hot region on a warm background. Therefore, it is reasonable to consider RC and NU at the same time. In this study, we assumed that the optimal reconstruction algorithm should satisfy the condition 0.9 < RC < 1.0 and NU < 10% simultaneously. The optimal criteria were marked in red color in Figure 3a,b. A high NU value means a noisy background in PET data. The tumor detectability would decrease in case of a high NU. According to the optimal criteria (0.9 < RC < 1.0 and NU < 10%) in the present study, OSEM2D (iteration 1) provided an optimal image parameter with an RC near 1 and low NU for both partially corrected and AC images. OSEM2D (iteration 1) provided an RC value of 0.92 and an NU of 7.0% for partially corrected and RC of 0.94 and NU of 7.1% for AC, respectively. However, when the AC and SC were applied, the RC value deteriorated to 0.70 for OSEM2D (iteration 1). 

### 4.3. Effect of Attenuation Correction and Scatter Correction

In this study, we focused on the correlation between NU and RC when no AC and/or AC/SC were performed. This was because AC and/or AC/SC was omitted during PET scanning of the mice When AC or AC/SC was performed, the tendency was similar to that of the partially corrected. 

### 4.4. Correlation between Recovery Coefficient and % ID/g in the Tumor Region

**Figure 6:**
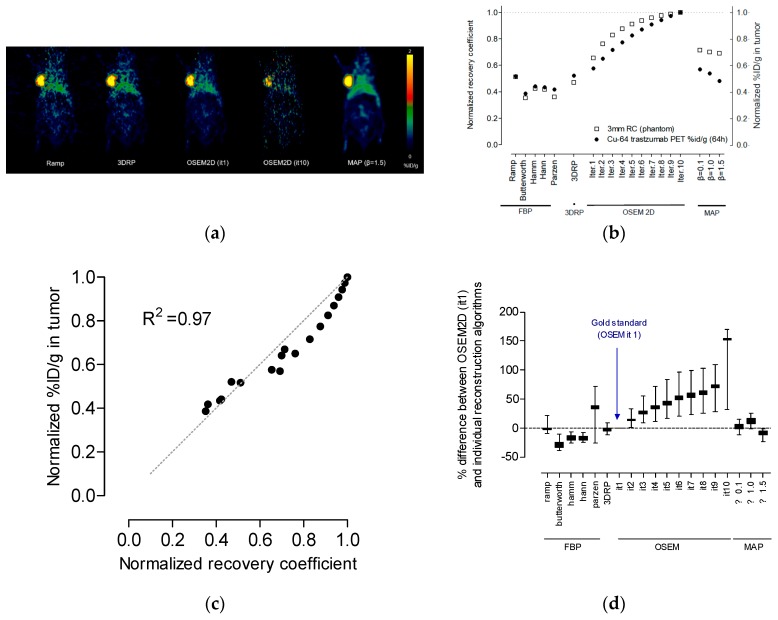
(**a**) Representative % ID/g image of Cu-64 trastuzumab PET using FBP (ramp filter), FBP-3DRP, OSEM2D (iteration 1), OSEM2D (iteration 10), and OSEM3D-MAP (β = 1.5). (**b**) Plot of normalized recovery coefficient (rod diameter is 3 mm) and normalized % ID/g in the tumor on Cu-64 trastuzumab PET. The recovery coefficient and % ID/g were normalized using each maximum value for comparison. (**c**) A plot of correlation between the normalized recovery coefficient (rod diameter is 3 mm) and the normalized % ID/g. The parameter was highly correlated (R^2^ = 0.97). (**d**) % difference between OSEM2D (iteration 1, the gold standard in this study) and individual reconstruction algorithms. The data was plotted using the averaged value of the difference for all organs and tumor regions. The data was plotted using the box and whisker plot. 3DRP: FBP-3DRP, MAP: OSEM3D-MAP.

To investigate whether the RC value calculated for phantom correlates with % ID/g calculated for Cu-64 trastuzumab, the RC value in this study was calculated for various reconstruction algorithms using the NEMA NU4 image quality phantom. RC from phantom study and % ID/g from Cu-64 trastuzumab PET were compared. For direct comparison with RC and % ID/g, RC and % ID/g were normalized with a maximum value of RC and % ID/g, respectively. The graphs of normalized RC and normalized % ID/g were plotted for various reconstruction algorithms. The ROI size for the tumor region was 0.066 cm^2^ (equivalent to a 3 mm rod for the RC phantom). Figure 6b shows the correlation between RC and % ID/g in the tumor region. Normalized RC and % ID/g in the tumor region were highly correlated (R^2^ = 0.97). RC was correlated with a value such as % ID/g or standard uptake value (SUV) on actual PET data sets. Thus, we could apply the optimal reconstruction algorithm, as determined from the proposed criteria in this study, for the actual calculation of dosimetry for Cu-64 trastuzumab PET.

### 4.5. Optimal Reconstruction Algorithm and Tumor Absorbed Dose

Table 1 presents the results of tumor dosimetry. According to the results, the value of the absorbed dose for the tumor ranged from 923 mGy/MBq to 1830 mGy/MBq. The difference between the maximum and minimum absorbed dose reached 98%. The minimum value of the absorbed dose for the tumor was obtained when FBP was used with Butterworth filter; the maximum value of the absorbed dose for a tumor was obtained when using OSEM2D with 10 iterations and 16 subsets. 

Table 2 presents the % difference between OSEM2D (iteration 1, the gold standard in this study) and individual reconstruction algorithms. When OSEM2D (iteration 1) was chosen as the optimal reconstruction algorithm based on the phantom study in this work (Figure 3a), OSEM2D (iteration 10) yielded 32.4% overestimated absorbed dose and FBP with Butterworth filter yielded 35.4% underestimated absorbed dose for the tumor. The maximum difference is marked in bold. 

There is the same tendency for all organs. For example, in the region of brain, OSEM2D (iteration 10) yielded 152.8% overestimated effective dose and FBP with the Butterworth filter provided 25.9% underestimated effective dose. In the region of the kidney, the difference was 154.7% overestimation for OSEM2D (iteration 10) and 20.9% underestimation for FBP with Butterworth filter. 

Figure 6c presents the averaged values of the % difference between OSEM2D (iteration 1, the gold standard in this study) and individual reconstruction algorithms. The box and whisker plot was used for Figure 6c. According to Figure 6c, we also found that there is a large difference between OSEM2D (iteration 1) and OSEM2D (iteration 10).

The limitation of this study is the potential bias of individual difference in dosage. This study essentially describes image quality characteristics as a function of reconstruction parameters for Cu-64. The data shown in Figure 6c were obtained from a single tumor uptake value. Therefore, the range in values represented was only due to differences in reconstruction.

## 5. Conclusions

OSEM2D (iteration 1) was determined for optimal reconstruction algorithms for minimizing the non-uniformity and for obtaining the highest RC. We suggested OSEM2D (iteration 1) for the calculation of the effective dose of Cu-64 trastuzumab on an Inveon PET scanner. 

## Figures and Tables

**Table 1:** Radiation dose estimates of the tumor and organs from mice injected with Cu-64 DOTA trastuzumab.

	FBP					3DRP	OSEM2D										OSEM3D-MAP		
	Ramp	Butterworth	Hamm	Hann	Parzen		Iter.1	Iter.2	Iter.3	Iter.4	Iter.5	Iter.6	Iter.7	Iter.8	Iter.9	Iter.10	β = 0.1	β = 1.0	β = 1.5
Tumor (mGy/MBq)	1200	923	1020	1060	1020	1250	1320	1340	1440	1480	1560	1620	1660	1710	1760	1830	1380	1500	1180
Organ (mSv/MBq)																			
Adrenals	0.0122	0.0094	0.0106	0.0104	0.0175	0.0121	0.0122	0.0140	0.0159	0.0175	0.0190	0.0206	0.0219	0.0227	0.0259	0.0911	0.0127	0.0141	0.0113
Brain	0.0327	0.0216	0.0222	0.0215	0.0556	0.0236	0.0263	0.0367	0.0466	0.0556	0.0642	0.0756	0.0775	0.0823	0.0899	0.3200	0.0233	0.0283	0.0207
Breasts	0.0036	0.0027	0.0031	0.0030	0.0053	0.0035	0.0037	0.0042	0.0048	0.0053	0.0057	0.0064	0.0066	0.0067	0.0077	0.0272	0.0036	0.0040	0.0033
Gallbladder wall	0.0107	0.0084	0.0093	0.0092	0.0147	0.0109	0.0105	0.0121	0.0135	0.0147	0.0158	0.0174	0.0180	0.0189	0.0211	0.0744	0.0110	0.0124	0.0096
Lower large intestine wall	0.0031	0.0023	0.0026	0.0026	0.0044	0.0031	0.0032	0.0036	0.0040	0.0044	0.0048	0.0053	0.0055	0.0058	0.0066	0.0231	0.0034	0.0038	0.0030
Small intestine	0.0041	0.0032	0.0036	0.0035	0.0057	0.0041	0.0041	0.0047	0.0052	0.0057	0.0062	0.0067	0.0071	0.0075	0.0084	0.0296	0.0043	0.0048	0.0038
Stomach wall	0.1060	0.0906	0.0947	0.0937	0.1230	0.1110	0.1010	0.1090	0.1160	0.1230	0.1300	0.1420	0.1440	0.1560	0.1820	0.6270	0.0993	0.1000	0.0925
Upper lower intestine wall	0.0046	0.0036	0.0040	0.0040	0.0063	0.0047	0.0046	0.0052	0.0058	0.0063	0.0069	0.0075	0.0078	0.0083	0.0093	0.0327	0.0048	0.0053	0.0043
Heart wall	0.0084	0.0064	0.0072	0.0071	0.0123	0.0084	0.0086	0.0097	0.0112	0.0123	0.0133	0.0148	0.0152	0.0155	0.0179	0.0629	0.0086	0.0094	0.0077
Kidneys	0.1890	0.1460	0.1650	0.1570	0.2680	0.1870	0.1800	0.2130	0.2420	0.2680	0.2930	0.2850	0.3390	0.3600	0.4040	1.4100	0.1830	0.2030	0.1720
Liver	0.0944	0.0746	0.0828	0.0821	0.1300	0.0979	0.0924	0.1070	0.1190	0.1300	0.1390	0.1550	0.1580	0.1660	0.1810	0.6440	0.0990	0.1130	0.0840
Lungs	0.1660	0.1240	0.1410	0.1390	0.2590	0.1630	0.1770	0.1980	0.2360	0.2590	0.2800	0.3140	0.3200	0.3220	0.3750	1.3200	0.1650	0.1790	0.1530
Muscle	0.0035	0.0027	0.0030	0.0030	0.0051	0.0035	0.0035	0.0041	0.0046	0.0051	0.0055	0.0061	0.0063	0.0066	0.0075	0.0264	0.0037	0.0040	0.0033
Ovaries	0.0031	0.0023	0.0027	0.0026	0.0045	0.0032	0.0032	0.0037	0.0041	0.0045	0.0049	0.0054	0.0057	0.0060	0.0067	0.0236	0.0035	0.0039	0.0031
Pancreas	0.0164	0.0127	0.0143	0.0141	0.0228	0.0162	0.0163	0.0187	0.0208	0.0228	0.0248	0.0276	0.0287	0.0297	0.0346	0.1210	0.0174	0.0190	0.0154
Red. Marrow	0.0038	0.0029	0.0033	0.0032	0.0056	0.0037	0.0038	0.0044	0.0051	0.0056	0.0062	0.0068	0.0071	0.0074	0.0084	0.0296	0.0039	0.0043	0.0035
Osteogenic	0.0031	0.0023	0.0026	0.0026	0.0047	0.0030	0.0031	0.0036	0.0042	0.0047	0.0051	0.0057	0.0059	0.0061	0.0070	0.0245	0.0031	0.0035	0.0028
Skin	0.0017	0.0013	0.0015	0.0014	0.0025	0.0017	0.0017	0.0020	0.0023	0.0025	0.0027	0.0030	0.0032	0.0033	0.0037	0.0131	0.0018	0.0019	0.0016
Spleen	0.4480	0.3260	0.3850	0.3820	0.6730	0.4120	0.4620	0.5380	0.6040	0.6730	0.7460	0.8620	0.8940	0.8990	1.0900	3.8000	0.5390	0.5960	0.4590
Thymus	0.0038	0.0029	0.0033	0.0032	0.0058	0.0038	0.0040	0.0045	0.0053	0.0058	0.0063	0.0070	0.0072	0.0073	0.0084	0.0296	0.0039	0.0042	0.0035
Thyroid	0.0014	0.0010	0.0011	0.0011	0.0021	0.0013	0.0014	0.0016	0.0019	0.0021	0.0023	0.0026	0.0027	0.0027	0.0031	0.0110	0.0013	0.0015	0.0012
Urinary bladder	0.1590	0.1140	0.1340	0.1320	0.2380	0.1600	0.1680	0.1940	0.2160	0.2380	0.2580	0.2890	0.2970	0.3160	0.3500	1.2300	0.1870	0.2120	0.1620
Uterus	0.0050	0.0037	0.0042	0.0042	0.0073	0.0050	0.0052	0.0060	0.0067	0.0073	0.0080	0.0089	0.0092	0.0097	0.0108	0.0381	0.0057	0.0064	0.0050

3DRP: FBP-3DRP.

**Table 2:** % difference between OSEM2D (iteration 1, gold standard in this study) and individual reconstruction algorithms.

	FBP					3DRP	OSEM2D										OSEM3D-MAP		
% Difference	Ramp	ButterWorth	Hamm	Hann	Parzen	3DRP	Iter.1 *	Iter.2	Iter.3	Iter.4	Iter.5	Iter.6	Iter.7	Iter.8	Iter.9	Iter.10	β = 0.1	β = 1.0	β = 1.5
Tumor	−9.5	−35.4	−25.6	−21.8	−25.6	−5.4	-	1.5	8.7	11.4	16.7	20.4	22.8	25.7	28.6	32.4	4.4	12.8	−11.2
Organ																			
Adrenals	0.0	−25.9	−14.0	−15.9	35.7	−0.8	-	13.7	26.3	35.7	43.6	51.2	56.9	60.2	71.9	152.8	4.0	14.4	−7.7
Brain	21.7	−19.6	−16.9	−20.1	71.6	−10.8	-	33.0	55.7	71.6	83.8	96.8	98.7	103.1	109.5	169.6	−12.1	7.3	−23.8
Breasts	−2.7	−31.3	−17.6	−20.9	35.6	−5.6	-	12.7	25.9	35.6	42.6	53.5	56.3	57.7	70.2	152.1	−2.7	7.8	−11.4
Gallbladder wall	1.9	−22.2	−12.1	−13.2	33.3	3.7	-	14.2	25.0	33.3	40.3	49.5	52.6	57.1	67.1	150.5	4.7	16.6	−9.0
Lower large intestine wall	−3.2	−32.7	−20.7	−20.7	31.6	−3.2	-	11.8	22.2	31.6	40.0	49.4	52.9	57.8	69.4	151.3	6.1	17.1	−6.5
Small intestine	0.0	−24.7	−13.0	−15.8	32.7	0.0	-	13.6	23.7	32.7	40.8	48.1	53.6	58.6	68.8	151.3	4.8	15.7	−7.6
Stomach wall	4.8	−10.9	−6.4	−7.5	19.6	9.4	-	7.6	13.8	19.6	25.1	33.7	35.1	42.8	57.2	144.5	−1.7	-1.0	−8.8
Upper lower intestine wall	0.0	−24.4	−14.0	−14.0	31.2	2.2	-	12.2	23.1	31.2	40.0	47.9	51.6	57.4	67.6	150.7	4.3	14.1	−6.7
Heart wall	−2.4	−29.3	−17.7	−19.1	35.4	−2.4	-	12.0	26.3	35.4	42.9	53.0	55.5	57.3	70.2	151.9	0.0	8.9	−11.0
Kidneys	4.9	−20.9	−8.7	−13.6	39.3	3.8	-	16.8	29.4	39.3	47.8	45.2	61.3	66.7	76.7	154.7	1.7	12.0	−4.5
Liver	2.1	−21.3	−11.0	−11.8	33.8	5.8	-	14.6	25.2	33.8	40.3	50.6	52.4	57.0	64.8	149.8	6.9	20.1	−9.5
Lungs	−6.4	−35.2	−22.6	−24.1	37.6	−8.2	-	11.2	28.6	37.6	45.1	55.8	57.5	58.1	71.7	152.7	−7.0	1.1	−14.5
Muscle	0.0	−25.8	−15.4	−15.4	37.2	0.0	-	15.8	27.2	37.2	44.4	54.2	57.1	61.4	72.7	153.2	5.6	13.3	−5.9
Ovaries	−3.2	−32.7	−16.9	−20.7	33.8	0.0	-	14.5	24.7	33.8	42.0	51.2	56.2	60.9	70.7	152.2	9.0	19.7	−3.2
Pancreas	0.6	−24.8	−13.1	−14.5	33.2	−0.6	-	13.7	24.3	33.2	41.4	51.5	55.1	58.3	71.9	152.5	6.5	15.3	−5.7
Red. Marrow	0.0	−26.9	−14.1	−17.1	38.3	−2.7	-	14.6	29.2	38.3	48.0	56.6	60.6	64.3	75.4	154.5	2.6	12.3	−8.2
Osteogenic	0.0	−29.6	−17.5	−17.5	41.0	−3.3	-	14.9	30.1	41.0	48.8	59.1	62.2	65.2	77.2	155.1	0.0	12.1	−10.2
Skin	0.0	−26.7	−12.5	−19.4	38.1	0.0	-	16.2	30.0	38.1	45.5	55.3	61.2	64.0	74.1	154.1	5.7	11.1	−6.1
Spleen	−3.1	−34.5	−18.2	−19.0	37.2	−11.4	-	15.2	26.6	37.2	47.0	60.4	63.7	64.2	80.9	156.6	15.4	25.3	−0.7
Thymus	−5.1	−31.9	−19.2	−22.2	36.7	−5.1	-	11.8	28.0	36.7	44.7	54.5	57.1	58.4	71.0	152.4	−2.5	4.9	−13.3
Thyroid	0.0	−33.3	−24.0	−24.0	40.0	−7.4	-	13.3	30.3	40.0	48.6	60.0	63.4	63.4	75.6	154.8	−7.4	6.9	−15.4
Urinary bladder	−5.5	−38.3	−22.5	−24.0	34.5	−4.9	-	14.4	25.0	34.5	42.3	53.0	55.5	61.2	70.3	151.9	10.7	23.2	−3.6
Uterus	−3.9	−33.7	−21.3	−21.3	33.6	−3.9	-	14.3	25.2	33.6	42.4	52.5	55.6	60.4	70.0	152.0	9.2	20.7	−3.8

* OSEM2D (iteration 1) reconstruction was regarded as the gold standard. All data were presented for partially corrected data, AC, and AC and SC data. 3DRP: FBP-3DRP.

**Figure A1:**
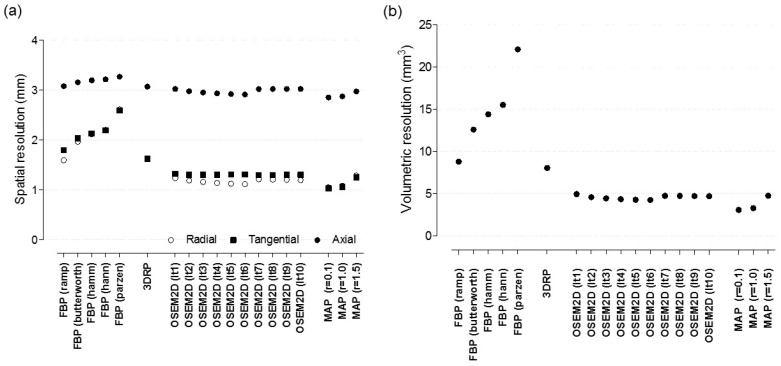
Spatial resolution of Cu-64 (**a**) radial, tangential, axial spatial resolution, (**b**) volumetric spatial resolution, 3DRP: FBP-3DRP, MAP: OSEM3D-MAP.

**Figure A3:**
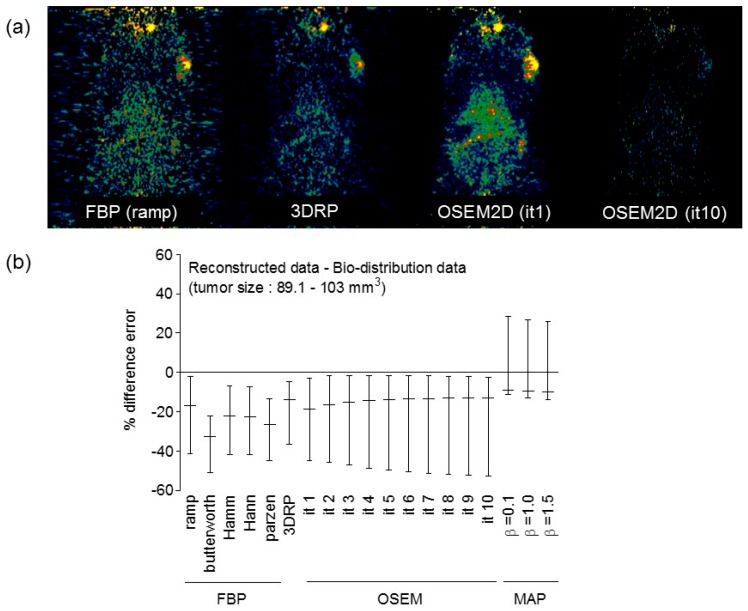
(**a**) representative PET of Cu-64 DOTA-anti-PD-1-antibody with FBP (ramp Figure 3. OSEM2D (it 1), OSEM2D (it 10), (**b**) %difference between reconstructed data and bio-distribution data. 3DRP: FBP-3DRP, MAP: OSEM3D-MAP.

**Figure A4:**
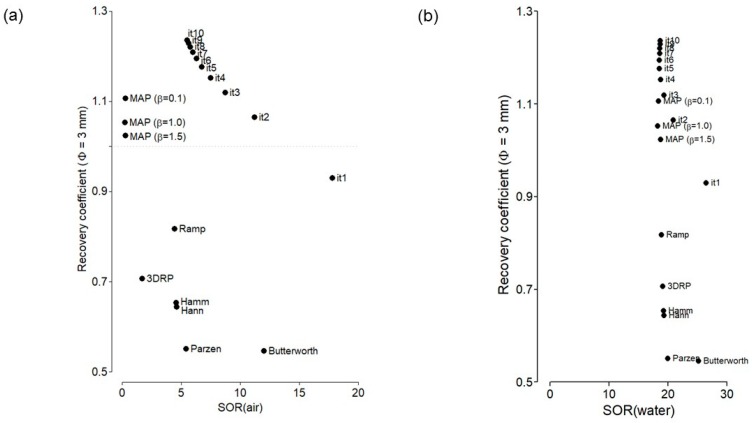
(**a**) SOR (air) vs. RC, (**b**) SOR (water) vs. RC. 3DRP: FBP-3DRP, MAP: OSEM3D-MAP.

**Figure A5:**
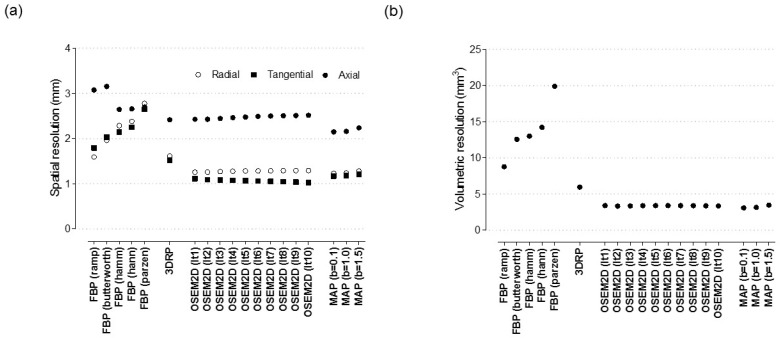
Spatial resolution of Zr-89 (**a**) radial, tangential, and axial spatial resolution, (**b**) volumetric spatial resolution. 3DRP: FBP-3DRP, MAP: OSEM3D-MAP.

**Figure A6:**
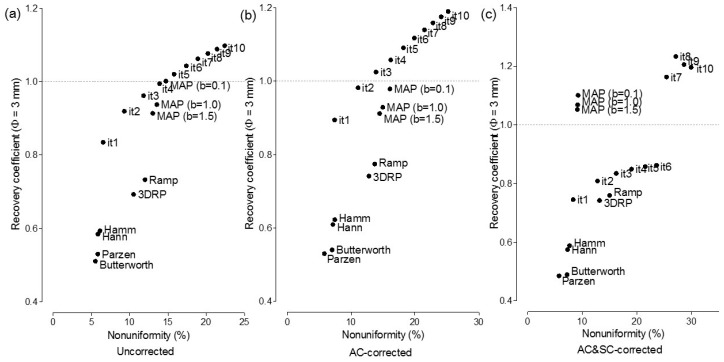
Plot of non-uniformity versus recovery coefficient (rod diameter is 3 mm) for various reconstruction algorithms, such as FBP, FBP-3DRP, OSEM2D, and OSEM3D-MAP reconstruction with filters. All data were presented for (**a**) partially corrected data, (**b**) AC, and (**c**) AC and SC data. 3DRP: FBP-3DRP, MAP: OSEM3D-MAP.

## Appendix A

### Appendix A.1. Spatial Resolution

To measure the spatial resolution, 1 mm^3^ sized point source (inner diameter 1.1 mm; wall thickness 0.2 mm) of Cu-64 was used. PET data was acquired for 5 min within an energy window of ~350–650 keV and a timing window of 3.432 ns. The list mode data were sorted into 3D sinograms and then reconstructed with various parameters. To determine the spatial resolution in radial, tangential, and axial directions, profiles through peaks in count distributions were drawn in two orthogonal directions in middle slices. For each profile, the full width at half maximum (FWHM) was measured by linear interpolation between adjacent pixels at one half or one tenth of their maximum values in profile. Axial profiles were also drawn to measure axial resolution. Volumetric resolution was calculated by multiplying resolutions for radial, tangential, and axial directions.

### Appendix A.2. The Effect of Reconstruction Algorithms with Various Filters

The effect of reconstruction algorithms was assessed for the FBP, 2D ordered subset expectation maximization (OSEM 2D), 3D reprojection algorithm (FBP-3DRP), and OSEM maximum a posteriori (OSEM3D-MAP) algorithms. In addition, the effect of filters in FBP was assessed for Butterworth, Hamming, Hanning, Parzen, ramp (cut off frequency: 0.5) and Shepp–Logan filters. For OSEM2D, the result of various iteration number from 1 to 10 was also assessed for the measurement of the effect of iteration number. Subset number was fixed to 16 for OSEM2D. For OSEM3D-MAP reconstruction, various smoothing factors (β) (0.1, 1.0, 1.5) were used with iteration number 18. The pixel size was 0.26 × 0.26 mm^2^. Normalization was applied, whereas AC and SC were not applied. The slice thickness values in all reconstructed images were 0.796 mm. Figure A1a,b shows the effect of reconstruction algorithms and filters on image resolution. The highest achievable volumetric resolution was 3.07 mm^3^ and it was obtained when OSEM3D-MAP (β = 0.1) was used.

### Appendix A.3. The Effect of Post-Smoothing with Various Gaussian Kernel

Figure A2 shows the result of OSEM2D (iteration 1) and OSEM3D-MAP (β = 1.5) data with application of Gaussian smoothing kernel. 1.5 mm, 3.0 mm, 4.5 mm, and 6.0 mm FWHM Gaussian kernel were applied to reconstructed PET data. Figure A2a,c show the plots of NU vs. RC (rod diameter is 3 mm) for OSEM2D (iteration 1) and OSEM3D-MAP (β = 1.5) with Gaussian kernel smoothed respectively. Figure A2b,d show PET images with Gaussian smoothing of phantom with OSEM2D (iteration 1) and OSEM3D-MAP (β = 1.5) reconstruction respectively. There was a tendency to decrease RC and NU as Gaussian smoothing kernel was applied. The percent difference of RC between without and with 6.0 mm Gaussian kernel smoothed was in 82% for OSEM2D (iteration 1) and 79% for OSEM3D-MAP (β = 1.5). The percent difference of NU between without and with 6.0 mm Gaussian smoothing was 68% for OSEM2D (iteration 1) and 69% for OSEM3D-MAP (β = 1.5).

### Appendix A.4. Comparison between Reconstructed Data and Bio-Distribution Data

To investigate the correlation between reconstructed data and bio-distribution data, we additionally performed PET and in vivo biodistribution study using Cu-64 DOTA-anti-PD-1 antibody data. Figure A3 shows the PET of Cu-64 DOTA-anti-PD-1-antibody and the correlation data reconstructed data and bio-distribution data. To acquire the PET/CT images, the Cu-64 DOTA-anti-PD-1-antibody (17.39–20.35 MBq, 400 μg) was administered to Hap-T1 cell-bearing hamsters when the tumor size reached 89.1–103 mm^3^. The immuno-PET/CT scanning (Siemens Inveon) was performed with an acquisition time of 15 min within an energy window of 350 to 650 keV at 60 h after injection of Cu-64 DOTA-anti-PD-1-Ab. In vivo biodistribution studies were also performed to evaluate the true uptake of Cu-64 DOTA-anti-PD-1-Ab in hamsters. Immediately after PET scanning, hamsters (*n* = 3) were euthanized by CO_2_ gas asphyxiation. The tumors were collected and weighed. The radioactivity of the tumor was measured as the total number of counts (counts per minute, cpm) using a Wizard2 gamma-counter (PerkinElmer). The activity data were represented as the percentage injected radioactivity dose per gram of tissue (%ID/g). PET data were reconstructed using various reconstruction algorithms and filters. %difference error between reconstructed data and bio-distribution data was calculated as follows: %difference error = (%ID/g of reconstructed data − D/g of bio-distribution data)/%ID/g of bio-distribution data. % difference error of OSEM (it = 1) ranged from −1.5% to −47.3%. This meant that the reconstructed value was lower than that of the bio-distribution data. When OSEM3D-MAP reconstruction method was used, the %difference error ranged from −13% to 28%. The positive value of the %difference error meant that the value of reconstructed data was higher than that of the bio-distribution data. This phenomenon was also observed in phantom data. This higher value was because of Gibbs artifact during OSEM3D-MAP reconstruction [25]. We found that the %ID/g of reconstructed PET data was lower than that of actual bio-distribution data. This phenomenon has also been found in a previous report [25]. According to the previous report, %ID/g of PET data was degraded by 20.9% compared to bio-distribution data [26]. This difference was possibly due to the effect of the partial volume on PET data. Because of limited spatial resolution on PET, the partial volume effect on the image of a tumor can degrade PET count. Another distinction is background uniformity. On the reconstructed PET data, image contrast would be important to detect tumor site. RC and NU would be deterministic factors in terms of detectability and image contrast. However, bio-distribution was free from partial volume effect and/or background activity because tumor or only the region being investigated was extracted and %ID/g was measured during bio-distribution experiment.

### Appendix A.5. Spill-Over Ratio vs. Recovery Coefficient

In this study, to determine the optimal reconstruction algorithm reconstruction, graphs between RC and NU were illustrated. In addition, we also plotted the graph between SOR (air, water) and the recovery coefficient in Figure A4a,b. SOR for water was >18% for all reconstruction algorithms and filters. RC was 0.92 and SOR for water was 26.4% when OSEM2D (it 1) was used.

### Appendix A.6. Assessment of Zr-89 PET Data in Terms of Spatial Resolution and SOR and NU

Zr-89 was also used for immune-PET. Therefore, we also assessed the spatial resolution and the relationship between the recovery coefficient and non-uniformity. The experimental procedures were the same as that of Cu-64.

### Appendix A.7. The effect of Reconstruction Algorithms with Various Filters

Figure A5a,b shows the result of spatial resolution. The highest achievable volumetric resolution was 3.09 mm^3^ and it was obtained when OSEM3D-MAP (β = 0.1) was used.

### Appendix A.8. Spill-Over Ratio vs. Non-Uniformity

Figure A6a–c shows RC for the 3 mm rods plotted against the non-uniformity when partially corrected, AC, and AC and SC, respectively. There was the same tendency between Cu-64 and Zr-89.
